# Is Electricity Life?

**Published:** 1891-04-11

**Authors:** 


### The Doctor's Armchair.

### IS ELECTRICITY LIFE?

No mortal knows either what electricity is or what life is. When, therefore, any person, for any purpose whatever, proclaims publicly that "Electricity is life," he at the same time proclaims himself to be at least an impudent fellow: if his knowledge be so small as to leave him unaware of the unsolved mysteries of life and electricity, he is ignorant as well as impudent. Undoubtedly, the most interesting of all the scientific inquiries of the present time is that which deals with electricity: with its potential wonders and its practical uses. The least scientific person can understand that electricity is a marvellous force. It is like fire and water in that it is a "bad master"; there are many reasons for hoping that it may also, like fire and water, prove to be a "good servant."

When the savage in his wanderings comes upon a strange animal or a new fruit, his first instinct is to wonder whether the fruit or the animal will be "good to eat"; and in like manner, when the civilised man discovers a new substance in chemistry or a new power in the wider laboratory of universal nature, he immediately bethinks him of testing whether it has got any life saving, pain destroying, vitalising, or other quality which will help him in his fight against mortality. Doctors have long been experimenting with electricity; and not only doctors but other persons. It must be admitted that the medical profession, as a whole, in Great Britain, and indeed all over the civilised world, is disappointed with the results obtained. No medical man of any science or sense doubts that electricity is an agent which may powerfully affect the human body and that in certain cases the results may be beneficial, But so little is certainly known of the nature of electric city, of the kind of action it exerts upon the body, and of the art of administering it with precision, that medical men in their daily work almost universally give it the cold shoulder.

But in these times the medical profession has to reckon with restless outsiders. It has perhaps always had that to do; only in more recent years the outsiders have frequently been persons of some education and scientific capacity, and have not seldom been inspired by public-spirited motives. The public recognise no monopoly in medicine. Doctors have certain vested interests, but they cannot establish their sole right to cure, still less to be the only persons to make curative experiments. In the case of electricity, every man who has a little knowledge of it feels that he would like to experiment in that most fascinating and profitable of all regions, the region of human diseases and disorders of health. The result is that electricity is more or less in the condition of a "suspect" in the estimation of many doctors, and that all who have anything to do with it, either professionally or unprofessionally, are obliged to be exceedingly circumspect if they wish to maintain an honest name.

But man, who is by nature impelled to strive for the mastery of every creature he sees, and of every force he recognises, can no more resist the impulsion to chain electricity and to put it to every possible kind of use, than he can extinguish the desire to master the ocean by building huge ships which may defy the mightiest storms. The electrical engineer uses electricity as a motor power, or as an illuminator; the doctor and the irregular pretender to medicine strive to bring it into the service of the healing art. What the public are concerned to know is whether electricity possesses any healing potency; and if it does, how that potency may be safely and advantageously utilised. Every honest contribution to our knowledge helps us to value with increasing correctness the therapeutic energy of this wonderful force, and also to adapt it to serviceable uses in the safest and most advantageous way.

At the Society of Arts last month Mr. Arthur Harries M.D., Associate of the Institute of Electrical Engineers, and Mr. H. Newman Lawrence, Member of the Institute of Electrical Engineers, jointly contributed a paper on "Electricity in relation to the human body, its dangers and its uses." It is plain that the proper persons to study what may be called "Medical Electricity" are doctors and electrical engineers. As the readers of the paper said: "For an electrician who is not also a medical man to prescribe electricity is utterly wrong; for a medical man who is not also an electrician to administer electricity is equally wrong; for one who is ignorant of both electricity and medicine to prescribe or administer an agent so powerful for good or evil, is criminal. The mysteriousness of electricity and its subtle methods of action render it peculiarly adapted to the purposes of the quack and the charlatan; while the indifference, suspicion, and conservatism of the medical profession, as a body, have hindered to an enormous extent the study and development of the uses of this valuable curative agent. The public have thus been thrown into the hands of men who, for the most part, know absolutely nothing, either of electricity or of the diseases they profess to cure. Still, truth must prevail; and we are convinced that however great the value of electricity in disease may be be now, its possibilities in the near future are far greater."

The whole position of "Medical Electricity" is stated in those few words. It is undoubtedly a most potent force; it is present in the air which the human body breathes; it is generated within the body itself in practically all physiological processes; the organism is susceptible to its power in a thousand ways. It is almost impossible, therefore, to conceive that there are no services of any kind which directly applied electricity can render to man. Reasoning *à priori*, we cannot but come to the conclusion that this most potent force, if rightly understood and applied, has in it elements of high therapeutic value. The problem is—how to use it so that it shall do the maximum of good and the minimum of harm. This problem has yet to be solved. As the writers of the paper show, we do not yet know how to manage such an elementary proceeding as the administration of a precise and accurate dose. And yet electricity is a more dangerous plaything than the poison of opium or of arsenic.

It is clear that a great advance in knowledge must be made before electricity can be of any considerable service in medicine. Electrical engineers must, in the first place, teach us to measure it as accurately as we can measure a grain of morphia, and to control the dose as precisely as we can control a dose of arsenic by means of the scales or the measuring glass. It is equally clear that untrained persons should be absolutely forbidden by law to use electricity therapeutically in any form. Everybody knows its power as a destructive agent; it is equally powerful as an agent of injury and damage when improperly applied. Members of the medical profession should alone be empowered to prescribe and supervise the administration of it, and they should be specially trained for the purpose. At present, thanks to the apathy of medical men and to the folly of the public, quacks and charlatans have electrical therapeutics almost entirely in their own hands. This is as discreditable to the profession as it is to the public. We owe it to ourselves, and to electricity, that so wonderful a force shall be rescued from its ignoble environment, and placed in that position of honour which it may rightly, and will ultimately, make its own.

